# Paradoxical HBsAg and anti-HBs coexistence among Chronic HBV Infections: Causes and Consequences

**DOI:** 10.7150/ijbs.55724

**Published:** 2021-03-11

**Authors:** Xinyi Jiang, Le Chang, Ying Yan, Lunan Wang

**Affiliations:** 1 National Center for Clinical Laboratories, Beijing Hospital, National Center of Gerontology; Institute of Geriatric Medicine, Chinese Academy of Medical Sciences, Beijing, P.R. China.; 2 Beijing Engineering Research Center of Laboratory Medicine, Beijing Hospital, P.R. China.; 3 Graduate School, Peking Union Medical College, Chinese Academy of Medical Sciences, Beijing, P.R. China.

**Keywords:** Hepatitis B surface antibody, Hepatitis B surface antigen, Mutation, Hepatocellular carcinoma

## Abstract

Hepatitis B surface antigen (HBsAg) and Hepatitis B surface antibody (anti-HBs) were reported simultaneously among Hepatitis B virus (HBV) infections. HBsAg is a specific indicator of acute or chronic HBV infections, while anti-HBs is a protective antibody reflecting the recovery and immunity of hosts. HBsAg and anti-HBs coexist during seroconversion and then form immune complex, which is rare detected in clinical cases. However, with the promotion of vaccination and the application of various antiviral drugs, along with the rapid development of medical technology, the coexistence of HBsAg and anti-HBs has become more prevalent. Mutations in the viral genomes, immune status and genetic factors of hosts may contribute to the coexistence. Novel HBsAg assays, with higher sensitivity and ability to detect mutations or immune complexes, can also yield HBsAg/anti-HBs coexistence. The discovery of coexistence has shattered the idea of traditional serological patterns and raised questions about the effectiveness of vaccines. Worth noting is that HBsAg/anti-HBs double positivity is strongly associated with progressive liver diseases, especially hepatocellular carcinoma. In conclusion, viral mutations, host factors, and methodology impacts can all lead to the coexistence of HBsAg and anti-HBs. This coexistence is not an indicator of improvement, as an increased risk of adverse clinical outcomes still exists.

## Introduction

Hepatitis B virus (HBV) infection is an enormous public health problem, seriously threatening a large number of undeveloped countries worldwide. In 2015, chronic hepatitis B infection (CHB) affected 257 million persons globally (3.5% of the world population) 1. Based on an epidemiology survey 2, China was suffering from the heaviest burden of HBV with 93 million HBsAg carriers, of which approximately 20 million were CHB patients. It was also estimated that nearly one million people died of HBV-related liver diseases every year with half coming from China 3.

During the infection, antibody response plays an important role in eliminating HBV particles and infected liver cells. Among the infected population, patients may naturally eliminate HBsAg and generate hepatitis B surface antibody (anti-HBs), while others may progress from chronic hepatitis to cirrhosis and hepatocellular carcinoma (HCC) 4, 5. Hepatitis B surface antigen (HBsAg) is the most important marker for the diagnosis of HBV infection. Anti-HBs is a specific protective antibody produced by the stimulation of exposed antigenic determinant, which can neutralize HBsAg in serum. The widely accepted goal of HBV therapy is seroconversion from HBsAg to anti-HBs. The dynamic transition may be the reason for the rare detection of HBsAg/anti-HBs coexistence. However, cases of simultaneous HBsAg and anti-HBs were frequently reported and difficult to be explained by the regular serological patterns. Arnold 6 first reported the concurrent anti-HBs and HBsAg with different subtypes both in serologic and fluorescence histologic studies. Though it has been over 40 years since they discovered this serological pattern, the underlying molecular mechanisms are still unclear and whether the coexistence is beneficial or detrimental to the recovery remained to be elucidated. In this review, we discuss the possible causes and clinical significance of the paradoxical HBsAg/anti-HBs coexistence and provide insights for unusual serological patterns.

## Mechanisms of coexistence

### Mutations in the viral genome

Using high-affinity monoclonal anti-HBs against separate determinants on HBsAg revealed the antigenic heterogeneity of HBV 7. Accordingly, antiviral therapy, vaccination, and natural immune pressure 8, 9 could account for these mutations.

Mutations are generally not limited to specific open reading frames (ORFs) and take place in all viral genes and regulatory elements 10. However, they tend to cluster together in specific regions, like the preS/S gene, reverse transcriptase region (RT) in the polymerase gene, the pre-core region, basal core promoter (BCP), and the X gene.

#### Mutations in the preS/S gene

**Figure 1:**
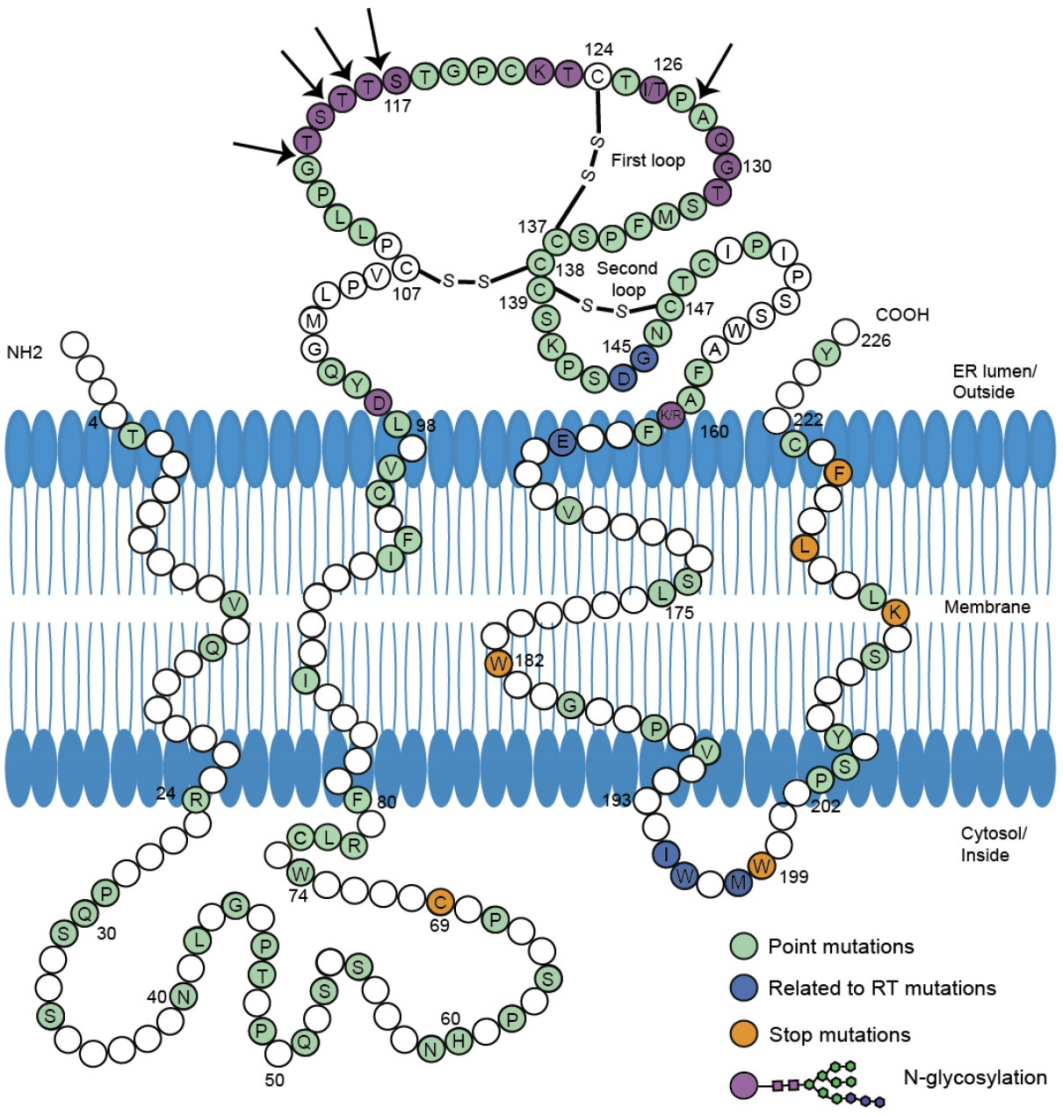
Mutations in S protein associated with HBsAg/anti-HBs coexistence. 226 amino acids of HBV small S protein were demonstrated in wild-type sequence with specific colors indicating different mutations. The conformation of the two major loops of the 'a' determinant is dependent on the presence of disulfide bonds (-S-S-). The proposed four transmembrane domains (TMD) and two cytosol loops were shown in the picture. The amino acid sequence of HBV S protein was retrieved from GenBank. The division of specific domains (TMD1:4-24 aa, TMD2:80-98 aa, TMD3:160-193 aa, TMD4:202-222 aa, MHR:99-160 aa) was referred to previous report^11^. According to the reference sequence ABE01542 (Genotype C, isolate S05014765), positions in white were not reported to mutate in HBsAg/anti-HBs coexistence. Positions in green were reported to occur in concurrent HBsAg/anti-HBs situation individually or jointly. Positions in dark blue were in connection with mutations in reverse transcriptase region of polymerase gene. Stop mutations were in orange and N-glycosylation in purple. Arrows were indicating insertions into amino acid sequence. All the mutations were listed in the Table S1.

Point mutations in the preS/S gene have been studied extensively in coexisting patterns (Figure 1). The major hydrophilic region (MHR) of HBsAg comprises the main conformational epitopes of viral particles 11. According to functional studies *in vivo* and *in vitro*, variations in the MHR can alter the antigenicity and immunogenicity of HBsAg, and diminish the reaction of anti-HBs 12, 13 (Table 1). Lada 14 found that mutation rates during coexistence group were 2.7-fold higher than during HBsAg alone. The rate of aa variability in the 'α' determinant of the MHR was also higher during coexistence (9.5% vs. 2.4%, p=0.009) than during HBsAg alone. In the first loop of 'α' determinant, mutations occurred in more positions in genotype C (Table S1). Another study found that the mutation rate at 126 aa (I126S/T/V) during coexistence was the highest and 6-fold higher 15. The high prevalence of I126 mutations in genotype C was consistent with what other studies found 16, 17.

In the second loop, mutations existed in all the positions. The G145R mutation was widely accepted as the most common immune-escape mutation 18. Variants with G145R could dramatically decrease the affinity for anti-HBs 19 and increase the compactness and stability of HBsAg by enhancing the rigidity of the 'a' determinant 20. However, G145R's detection varied in different genotypes. In genotype C, G145R during coexistence or G145A during HBsAg alone was rarely detected 15, 21. Among patients with genotype B, G145R occurred in coexistence group but was not detected in HBsAg alone group 17. G145R in genotype B and I126 mutations in genotype C can thus be the representative MHR substitutions in HBsAg/anti-HBs coexistence.

Glycosylation, insertion and early termination in S proteins influence the expression, secretion and recognition of HBsAg 29, 37. Glycosylation reduces the affinity between anti-HBs and HBsAg by decreasing HBsAg antigenicity and impairing the neutralization ability of anti-HBs. N-glycosylation occurred more frequently during coexistence than during HBsAg alone (47/216 vs. 1/182, p<0.001) 23. Position 129-131 aa displayed highest N-glycosylation rates. N-glycosylation insertions clustered around position 112 to 116 aa and some large insertion patterns shown in Table S1 were first reported during coexistence 22, 34, 38. The insertions may affect biological properties and antigenicity of HBsAg to a greater extent. Stop mutations W182* and W199* 24 have been reported in the transmembrane domain (TMD) near the C-terminal of S proteins. Stop codons and the resulting early termination in HBsAg lacked the major epitopes recognized by specific antibodies, which could favor persistent viral replication and prolonged mutant HBsAg existence in the presence of anti-HBs.

**Figure 2:**
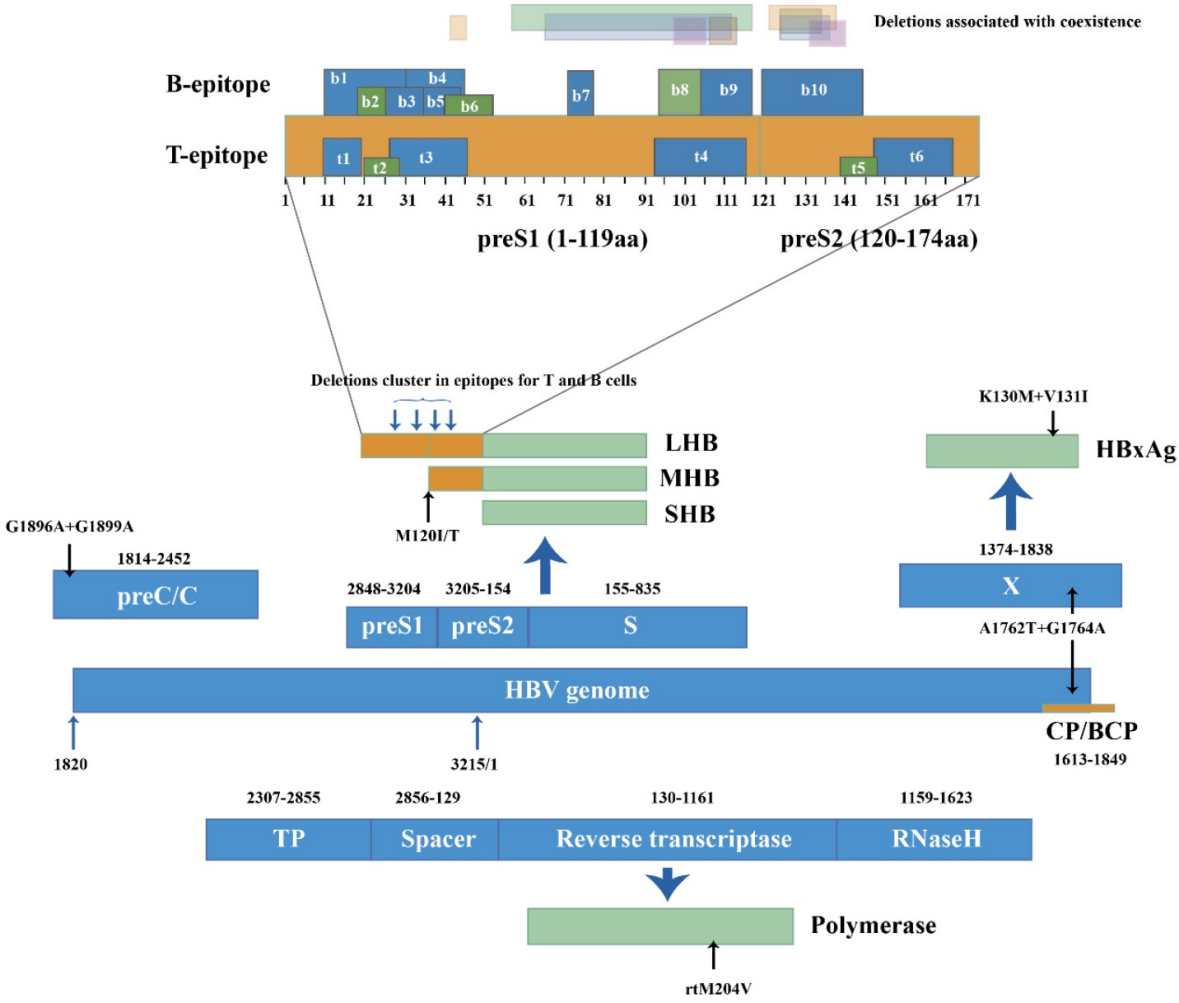
HBV genome (1-3215 nucleotide, nt) was represented in linear diagram. Different open reading frames and their encoded viral proteins were connected with bold blue-filled arrows. Light black arrows pointed out specific mutations in nucleotide level and amino acid level, respectively. PreS/S gene encoded three products: Large-HBsAg, Middle HBsAg and Small HBsAg (LHB, MHB and SHB). B and T epitopes in the preS products were numbered from N to C terminal. Translucent bars in different colors represented preS1 and pre S2 deletion patterns in HBsAg/anti-HBs coexistence.

Mutations and deletions in the epitopes for T and B cells can also explain the coexistence situation. In genotype C, mutations I92T, F93C and C95W occurred in cytotoxic lymphocyte (CTL) epitope of S proteins and led to higher aa variation compared to HBsAg alone group (p=0.002) 34. Huang 21 reported coexistence group yielded more deletions than HBsAg alone group at the T and B cell epitopes of the preS gene (25.0% vs. 3.6%). The deletions clustered within the C-terminal of the proteins encoded by preS1. Deletions specific to preS2 were located at the N-terminal of preS2 proteins, covering two epitopes for T cells and one epitope for B cells 27, 39 (Figure 2). Moreover, preS2 deletions were only found in coexistence group from genotype C-infected patients 21. The mutations and deletions appear at C-terminal of preS1 proteins and N-terminal of preS2 proteins, impairing the recognition sites for immune cells and preventing CTL activation. Because the immune system produces few or even no active anti-HBs, immune-escape mutants survive and coexist with anti-HBs.

#### Mutations in the transcriptase region

Studies on coexistence due to polymerase gene mutations are limited and are mainly focused on the RT region. Considering the overlapping structure of the S gene and RT region, mutations within S gene may account for changes in the P genes 40. A significantly higher aa variability was observed within RT regions overlapping the MHR when compared between coexistence group and the HBsAg alone group (1.14 vs. 0.17, p=0.003), and mutations rtI16T, rtD134E, rtS135T/Y/N, rtR138N/K, rtN139Q and rtR/W153Q/K/H in genotype A, B or D were significantly more frequent in the coexistence group (p<0.05) 29, 31. These mutations were harboring at the functional finger subdomain of the enzyme 41. Other than rtI16T, the other mutations had corresponding HBsAg mutants located in the 'α' determinant. The dual mutations rtR153Q/sG145R, which occur in separate genes can inhibit HBV replication by affecting polymerase activity 42 and greatly decrease the neutralizing ability of anti-HBs by altering the immunogenicity of HBsAg.

Antiviral treatments may stimulate immune selection pressure and induce viral mutation acceleration. rtL180M and rtM204V appeared in a patient who was undergoing anti-HBV therapy when HBsAg/anti-HBs coexistence was first detected 29. Several RT mutations corresponding to HBsAg mutants including sE164D, sI195M, sW196S, sM198I and sE164D+sI195M were also selected during lamivudine treatment and existed in coexistence group 33, raising the possibility that therapy-resistant mutants possess antigenically distinct HBsAg proteins that cannot be neutralized by anti-HBs. Another vaccine escape mutation that occurred in coexistence was rtA181T/sW172*, which had a dominant negative secretion effect and led to clearance of wildtype HBV antigen from the serum 43. This mutation may help vaccine escape mutants coexist with the anti-HBs induced by vaccine 44, 45. Although treatment-induced mutations in RT regions did not display high frequency in coexistence group, their concurrence raised questions about the effectiveness of antiviral drugs and vaccines.

#### Mutations overlapping the preC/BCP and X genes

BCP and pre-core mutations are prevalent in HBV chronic infection and associated with the severity of liver diseases 46. During coexistence, point mutations G1742A, T1753C, A1755C, T1768A, C1773T, A1775G, T1802C and A1846T occur in the BCP region while G1896A and G1899A occur in the pre-core at the nucleotide level 24. The BCP double mutations A1762T and G1764A are found more frequently in coexistence patients than in HBsAg alone patients (p=0.012) 34. The dual mutations can enhance viral genome replication and may partially resist neutralization of anti-HBs 47. A1762T+G1764A at the nucleotide level can also lead to K130M+V131I at the HBxAg coded by the X gene 36, which is CTL's target and can mediate the immune recognition of CTL. Mutations and early termination in HBxAg may not stimulate CTL activity and can slow down the clearance of HBsAg, thus generating uncommon serological patterns. Mutations in the preC/BCP and X region associated with coexistence were reported in limited samples. To investigate coexistence and advanced-disease-related mutants, more studies with larger sample sizes must be carried out.

### Immune status and genetic factors of hosts

Adverse immune status of hosts can lead to superinfection of different HBV strains when no mutation exists in the HBV genome. Breakthrough or reactivation of HBV may occur and facilitate the heterologous HBsAg/anti-HBs coexistence 18, 29, 48.

HBsAg subtype determinants had diverse physical and chemical properties, and infection by one subtype generated incomplete cross-immunity, favoring the mixed infection of different subtypes. Thus, heterologous subtype-specific antibody in coexisted serological pattern is commonly produced through the infection with different HBV serotypes. Purcell 49 vaccinated several seronegative chimpanzees with prepared antigen of subtypes ayw or adr, and infected them with the ayw subtype HBV six months later. The chimpanzees inoculated with the same subtypes did not show evidence of developing HBsAg or hepatitis. However, one of the chimpanzees vaccinated with adr subtype was infected, indicating the absence of complete protection between subtypes. Japanese scientists then discovered HBsAg and anti-HBs of different serotypes in chronic HBV carriers 50. Reinfection with a second subtype may be the reason as anti-HB against a different subtype than that of the circulating HBsAg was found 51. In a neutralization experiment, 74.3% (26/35) anti-HBs in coexistence patients could not neutralize the HBsAg in their serum and 28.6% (10/35) of HBsAg in the same group could not be fully neutralized by the anti-HBs from vaccinated persons 17. Moreover, antibodies from four patients could not neutralize the three major HBV serotypes (adw, adr and ayw) in China 52. These experiments are strong evidence that HBsAg and anti-HBs develop from different serotypes and the coexistence pose an underlying threat to general populations, even to vaccinated persons.

HBV reactivation associated with immunosuppressive conditions is another possible mechanism 53. The concurrent detection of HBsAg and anti-HBs is a possible signal of HBV reactivation, which occurs mainly in HBV resolved patients who received chemotherapy or immunosuppressive therapy without antiviral prophylaxis. Patients with resolved HBV infection often have negative HBsAg, positive hepatitis B core antibody (anti-HBc) and protective anti-HBs. Wang 48 emphasized that active viral replication or occult infection still occurs in these patients, and anti-HBs offers no protection against the virus. In patients with HBV reactivation, viral replication would accelerate again to produce a large amount of HBsAg in a short time, leading to the coexistence situation and therapy-related HBV reactivation. The reactivation may finally lead to lethal consequences such as fulminant hepatitis or hepatic failure 54.

Host genetic factors could play a role in the clearance of HBV and influence the occurrence of anti-HBs in chronic HBsAg carriers 55. The human gene oligoadenylate synthetases 3 (OAS3) may degrade viral RNAs and restrict the HBV replication, which was related to HBsAg/anti-HBs coexistence through a burden study. The overexpression of mutated OAS led to inactivation of IFN-induced OAS/RNase L pathways; and the resulting insufficient antiviral effects may cause failed clearance of HBsAg. Although further functional study of OAS3 gene is needed to evaluate its contribution to the coexistence group, host factors' influence on antiviral responses cannot be overlooked.

### Impact of methodology

The improvement of diagnosis methods rather than faulty methodology in HBsAg or anti-HBs detection could primarily account for the coexistence 34. Although HBV genetic variability poses threats against the current assays, in-depth serological and molecular researches contribute to the development of the reagents. Over the years, the sensitivity and specificity of assays have greatly improved. Huzly 56 compared nine commercial assays for anti-HBs quantification. An accurate measurement for a series of diluted international standard was achieved by most of the systems, including both enzyme immunoassays (EIAs) and chemiluminescent immunoassays (CIAs). After evaluating and comparing the performance of available anti-HBs reagents 57, new assay formats were designed to optimize their specificity 58.

Qualitative assays for HBsAg had the ability to detect classical mutations in the MHR 59, including point mutations at positions 123, 129, 133, 144, 145 and P142L/S+G145R double mutations. Lou 60 reported an ultra-sensitive Architect assay for the detection of HBsAg, which can detect up to 97 designed mutations covering the key positions in S proteins. From qualitative detection to quantitative determination, the limit of HBsAg reached the boundary of 50 mIU/ml 61. Pancher 62 demonstrated favorable overall performance of quantitative assays for HBsAg from coexistence patients and that the circulating anti-HBs did not influence HBsAg quantification. Moreover, in sandwich CIAs applying both polyclonal and monoclonal antibodies, no specific protein pattern can impair the quantification which was supported by all the assays 63.

Development of highly‐sensitive assays was a trend for future HBsAg quantitation. A novel chemiluminescent enzyme immunoassay (CLEIA) was designed for quantitative HBsAg, which used monoclonal antibodies targeting both common 'a' determinant and loop inside the lipid bilayer 64. The prototype of the two-step sandwich assay was further applied on Lumipulse system (Fujirebio Inc). The sensitivity of Lumipulse HBsAg-HQ was 5 mIU/ml, approximately 10-fold higher than previously reported assays. By disrupting HBV particles, dissociating HBsAg from HBsAg/anti-HBs complexes and denaturing epitopes to a linear form, thus fully exposing the outer and inner epitopes of HBsAg. As a result, in 26 HBsAg seronegative patients, Lumipulse HBsAg-HQ assay detected HBsAg in 4 patients with a protective anti-HBs concentration (over 10 mIU/ml), 3 of whom had no HBsAg escape mutations 65. The highly sensitive CLEIA Lumipulse HBsAg-HQ is suitable for HBV monitoring, which may raise the questions of vaccine effectiveness and disrupt the awareness of traditional serological patterns.

The false positive of serological markers caused by sample storage, specimen handling, as well as reagent usage should also be dealt with 53, as the detection can be influenced by contamination, interfering substances and the loading amount of plasma 66. The results of different studies need cautious analysis and comparisons 67, 68. The identification of the coexistence group in clinical experiment needs re-examination and the use of reliable reagents is essential to reduce errors caused by reagents and operating process. Detection of HBV serum markers can be combined with HBV DNA, liver function and other clinical symptoms. A dynamic and comprehensive analysis can provide a dependable basis for clinical diagnosis and treatment.

## Clinical characteristics and significances in coexistence

### Clinical characteristics and advanced liver diseases in coexistence

The prevalence of simultaneous anti-HBs and HBsAg positivity was found in 2.4%-5.8% of chronic HBV infections in mainland China from 2007-2019 (Table 2). However, the higher prevalence rates (21%) were obtained in Japan and Singapore in 1996, which might overestimate the occurrence of concurrent HBsAg/anti-HBs due to limited sample size and inclusion of acute HBV infection.

The coexistence of HBsAg and anti-HBs might be associated with important clinical concerns and this profile could be linked to advanced fibrosis, hepatocellular carcinoma (HCC) and liver failure 69. Among 92 patients with simultaneous HBsAg/anti-HBs occurrence in Korea, 6.5% suffered from liver cirrhosis and 2.2% were diagnosed with HCC 70. With the follow-up of 1042 non-HCC patients for a median 4.3 years, 13.7% (10/73) patients with coexistence developed HCC, which was higher compared with HBsAg alone group (6.9%, 67/969) 71. Coexistence of HBsAg/anti-HBs independently increased the risk of HCC in CHB infection 71, 72. There is a 3.08-fold (95%CI: 1.26-7.55) higher risk of HCC than the HBsAg alone patients 53. Characteristics in HBsAg/anti-HBs coexistence such as age over 50 years and abnormal ALT levels were also risk factors for HCC, which was linked to a long history of HBV infection with advanced liver fibrosis and active inflammation 29, 72. Serum PIVKA-II, better correlated with portal vein invasion, larger tumor size and recurrence of HBV-associated HCC 73, was also observed with higher levels in coexistence group 69.

### Mutations and HCC

Mutations in HBV ORFs and the encoding mutated proteins connected the progressive liver diseases with the concurrent HBsAg/anti-HBs. HCC patients with HBsAg/anti-HBs coexistence had a significantly higher frequency of N-glycosylation mutations in the first loop of S proteins compared to non-HCC patients (22.4% vs. 8.0%, p<0.01) 22. Longitudinal observation revealed these N-glycosylation mutations were detected 1-4 years prior to HCC occurrence, which was also found in other mutation patterns, the preS deletions 72 and T1762+A1764 double mutations 82.

PreS/S deletions in HCC were consistent with those from coexistence group, which displayed lower prevalence in HBsAg alone group. These deletions in HCC patients collected from their serum or liver tissues 83, clustered in the immune epitopes for T and B cells, especially in the C-terminal of preS1 and N-terminal of preS2, affecting the recognition of virus by immune cells 72. These deletions can trigger a protein kinase C (PKC) dependent activation of signal transduction cascade and induce the AP-1 and NF-κB transcription factors to enhanced hepatocytes proliferation 84, demonstrating the mutant proteins encoded by preS/S can be the transcriptional activators facilitating the invasions of the tumors 85. Mutations in the preS2 initiation codons affect the secretion of the virus and cause the accumulation of viral proteins in host cells. The mutation at the site 120 in genotype B, utilizing a different pathway in liver disease progression that involves high expression of NF-κB subunit p50 28, activated NF-κB as one of the mechanisms in inducing advanced liver disease. PreS1 and preS2 deletions in ground glass hepatocytes is characterized by an abnormal retention of mutant large and middle surface proteins (LHBs and MHBs) in endoplasmic reticulum (ER) 86. Deletions in preS from coexistence patients may generate truncated LHB and MHB. The mutants may also retain in the ER and induce ER stress signals as well as oxidative stress responses, upregulating COX-2 and cyclin A to induce cell cycle progression and generating a large amount of reactive oxygen species (ROS). Genomic instability in hepatocytes 87 and prolonged hepatocellular injuries can initiate a programmed response characterized by inflammation, regenerative hyperplasia, transcriptional deregulation and aneuploidy 72. Such effects can lead to the development of HCC, as proven in studies with transgenic mice 83.

BCP double mutations A1762T+G1764A with a significantly higher frequency in the coexistence group functioned as HCC predictors in HBV genotype B or C carriers 35, 88. This double mutation shared the common pattern including the codon 'AGG' with the sense strand sequence of p53 35. Aflatoxin can bind and induce mutations in this codons of p53 during the pathogenesis of HCC 89, suggesting A1762T+G1764A might be a mutational hot spot targeted by aflatoxin or some other chemical agent. Moreover, A1762T+G1764A harbored in X gene led to L130M+V131I substitutions in HBxAg (Figure 2). Involved in many intracellular signal pathways, HBxAg was closely associated with cell proliferation and cell apoptosis 90 and the mutant proteins displayed stronger tumorigenic effects than the wildtype. In Huh-7 cell lines 91, the double mutation accelerated fibrosis and cirrhosis via inducing ROS production and mitochondrial depolarization. Their contribution to HCC may be direct involvement in host cell proliferation and hepatocarcinogenesis via altering expression of cell cycle regulatory genes. Variant with L130M+V131I mutations may also exert a synergistic effect in accelerating the progression to HCC 92, which was recently reported to promote HCC progression by activating AKT/FOXO1 pathway and inducing more severe inflammation in liver via arachidonic acid metabolism 93.

### Dilemma for treatment

In the clinical cases, Galati 94 reported the dilemma of treatment when HBsAg and anti-HBs were coexisting. Interferon therapy as well as several nucleotide analogues (NAs) specific for HBV failed to achieve virologic responses. Only with the combination of second-generation NAs did the patient finally experience HBsAg loss. In another case 95, after antiviral treatment with entecavir for eight years, the female patient showed the serological profile characterized by high titers of anti-HBs and the consistent levels of HBsAg compared to three year ago. Although HBV DNA was undetectable, the patient had the risk for HBV reactivation and further treatment went into a dilemma. The irregular HBV DNA were also reported in different studies with the coexistence situation 18, 96. Antiviral therapies may decrease serum HBV-DNA levels, concealing the influence of serum HBV DNA levels on HCC development 71. Reversely, HBsAg/anti-HBs coexistence patients with relatively high viral loads suggested active virus replication and raised the problem of HBV transmission 14, 48. For HBsAg/anti-HBs coexistence patients, the irregular viral load may mislead the treatment and risk evaluation.

Under some special conditions, the HBsAg/anti-HBs coexistence may increase complexity for treatment and predict poor prognosis. Approximately 10-15% of hepatitis C virus (HCV)-infected people and about 10% human immunodeficiency virus (HIV)-infected people were diagnosed with HBV 97, 98. During coinfection, interactions between the two viruses may change the natural history of both mono-infections. Reactivation of HBV can occur in HIV-driven immune suppression and HCV-related direct-acting antivirals therapy, and then facilitate coexistence of HBsAg and anti-HBs. Moreover, the coexistence may also occur in the presence of hyperimmune function. As HBV is a common comorbidity among rheumatic patients, HBV infection may reactivate in patients with autoimmune inflammatory rheumatic diseases when they take immunosuppressive drugs or undergo biological therapies 99, 100. The HBV infection needs surveillance, and markers like HBsAg and anti-HBs need to be detected before and after the treatment of co-infected diseases.

### Clinical significance and monitoring suggestions

A certain proportion of population may be confronted with coexistence of HBsAg and anti-HBs. The emergence of anti-HBs does not demonstrate that HBsAg can be completely or effectively eliminated 96, the virus can replicate continuously with the presence of anti-HBs. While some individuals are prone to the coexistence in clinical practice. Firstly, patients who have risks of HBV breakthrough or reactivation are in need of follow-up and monitoring. They may undergo immunosuppression condition because of immunosuppressive therapies and/or coinfection with immunodeficiency diseases. Secondly, although HBV vaccines were delivered to newborns in many countries, the vaccine anti-HBs had lower binding capacity to mutant HBsAg 17 and anti-HBs levels declined with age 101, some young individuals still had risks of HBV infection, especially those born to HBsAg positive mothers. Thirdly, in occult HBV infection (OBI), HBsAg was undetectable with the existing detection methods. However, some specific mutations in preS/S gene were found in anti-HBs positive OBI samples 102 and might be adaptive substitutions selected under immune pressure. These are risk factors for virus reactivation under anti-HBs selection and changes of serological markers in OBI patients is worthy of clinical attention.

To further validate and monitor HBsAg/anti-HBs coexistence, diagnosis procedures and surveillance measures are required. Although no standard diagnosis procedure is available, what we can do is to apply reliable and sensitive detection methods for both HBsAg and anti-HBs in the first place, which can avoid false positive or false negative results. The results should also be combined with other markers such as HBV DNA and liver function indicators. Quantification of HBV DNA, along with genotyping and mutation analysis can be helpful to identify the virus, whether it's a wild-type or mutant. After ensuring the accuracy of our test results, monitoring measures are specific for different groups of people. For pregnant women and infants, we suggest to screen all pregnant women for HBsAg and provide immunoprophylaxis to infants of HBsAg-positive mothers. Moreover, routine immunization of HBV vaccine is needed for all infants. For adolescents and adults, especially those at a high risk of HBV infection (injection drug users and individuals who participate in high-risk sexual behaviors), we suggest to screen them for both HBsAg and anti-HBs periodically. If the anti-HBs titer is below the protective level, a booster dose vaccine is needed to strengthen immunity. For chronic HBV-infected patients and OBI patients, enhanced surveillance of liver function and serological indicators is urgently needed. They should go through longer follow-up of their serological patterns, and the physicians should pay close attention to the changes in the HBsAg and anti-HBs levels.

## Conclusions

**Figure 3:**
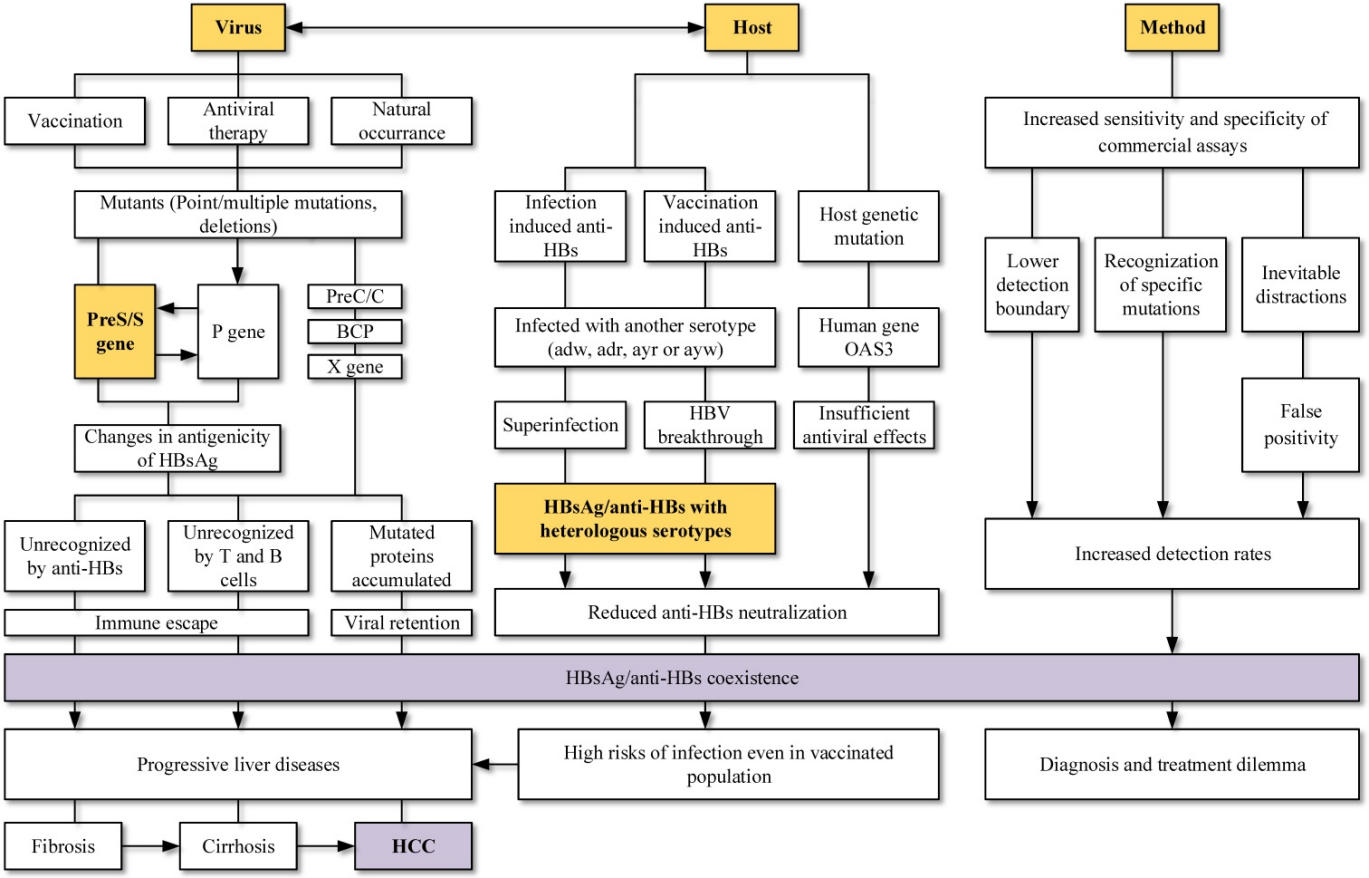
Viral mutation host factors, the interaction between viruses and hosts, as well as screening method were connected to the HBsAg/anti-HBs coexistence. Mutations in viral genome, especially mutations in MHR and deletions in preS gene, were widely studied and reported to change the antigenicity of HBsAg. Superinfection and breakthrough infection can lead to simultaneous detection of serotype-specific HBsAg and anti-HBs. Changed epitopes cannot be recognized by immune cells and suppressed immune system allowed the complex infections. Coexistence is not the indicator of improvement and an increased risk of adverse clinical outcomes still exists.

HBV is related to large-scale morbidity and mortality, bringing heavy burden to the developing countries. Knowledge of paradoxical serological patterns can be helpful for the control and eradication purpose of HBV. Clinical laboratories encounter HBsAg and anti-HBs double positivity more frequently at present. Its possible causes and clinical significance (Figure 3) are worth to be considered and further explored.

Mutations in different ORFs of HBV is a possible and convincing interpretation for the HBsAg/anti-HBs coexistence. Point mutations in the preS/S gene, RT region of polymerase gene, as well as preS deletions can both change the stability and immunogenicity of HBsAg, favoring the selection of immune escape variants and decreasing the affinity with anti-HBs. Vaccination, antiviral therapy and even HBV chronic infection without any treatment can lead to accumulation of those HBV variants. Relationship between virus and host should also be considered. Superinfection and breakthrough infection are associated with the presence of heterologous subtype-specific anti-HBs rather than mutations in the gene region, particularly in the clinical settings of immunosuppression. The improvements in screening methods may lead to the detection of lower HBsAg levels, mutants and immune complexes, increasing the detection of coexistence. Keep updating the explanations for different serological patterns is necessary.

Lastly, the coexistence is not the indicator of recovery and an increased risk of adverse clinical outcomes still exists. Patients with HBsAg/anti-HBs coexistence may progress into liver cirrhosis, HCC and liver failure. Vaccinated persons had risks of infection and the coexistence of HBsAg/anti-HBs may lead to a dilemma for diagnosis and treatment. More studies about the coexistence of HBsAg/anti-HBs should be carried out and corresponding countermeasures shall be taken by both researchers and physicians.

## Supplementary Material

Supplementary figures and tables.

## Figures and Tables

**Table 1:** Mutations associated with HBsAg/anti-HBs coexistence

Regions		Mutation patterns	Possible mechanisms of coexistence	Representative mutations	
				More frequently/only found in HBsAg/anti-HBs coexistence	Associated with progressive liver diseases
S gene (encoding small HBsAg)	MHR (99-160 aa)	Point or double mutations (including glycosylation)	Alter immunogenicity of HBsAg and reduce reactivity for anti-HBs induced by the recombinant HBV vaccine	Genotype B: sG145R 17 Genotype C: sI126S/T 15	Genotype B: sT131N+sM133T Genotype C: sT116N, sQ129N, sT131N, sG130N+sT131N, sG130N+sT131I 22, 23
		Inserted mutations or deletions	Change the antigenicity of HBsAg in a greater extent	Genotype B: s126-127 “SARIVNTT” insertion Genotype C: s112-113 “KNA” insertion, s114-115 “NTSTT” insertion, s115-116 “INGTST” insertions, s116-117 “STGR” insertions 22, 24	Genotype C: s112-113 “KNA” insertion, s114-115 “TTN”/”NTSTT” insertion, s115-116 “INGTST” insertions; sT113N+ deletion in 114-116 aa 22
	Outside of MHR	Point mutation and stop mutations	Mutations in cytotoxic lymphocyte epitope affect the recognition of immune cells and affect binding to anti-HBs; Early stop mutation in the transmembrane domain influence the stability and secretion of HBsAg, affect the structure of virus-like particles and the binding of anti-HBs	sC69stop, sW182stop 17	Genotype C: sW182stop 25
preS/S gene (encoding large and middle HBsAg)	preS1 (1-119 aa) 4 epitopes for T cells; 9 epitopes for B cells	Deletions and stop mutations	Interrupt viral surface epitopes and evade immune-surveillance; Mutations are unable to be recognized by immune cells		Genotype B: Deletion in 98-106 aa Genotype C: Deletion in 57-117 aa, 66-112 aa, 107-113 aa^21^, ^26^
	preS2 (120-174 aa) 2 epitopes for T cells; 1 epitope for B cells	Deletions and stop mutations	Mutation at the epitope for T and B cells influence the recognition of immune cells; Alter the expression and secretion of HBV envelope proteins and result in intracellular accumulation of mutant proteins		Genotype C: Deletion in 125-135 aa, 122-139 aa, 125-138 aa, 132-141 aa^21^, ^27^
		Initiation codon mutations	Affect the secretion of the virus and cause the accumulation of viral proteins in host cells; Mutant proteins are unable to be recognized by immune cells		Genotype B: M120I/T/V^24^, ^28^
P gene (Polymerase)	RT region (1-344 aa)	Point or multiple mutations	Corresponding S gene mutants possess antigenically distinct HBsAg proteins which decrease HBsAg antigenicity and impair the neutralization ability of the antibodies	Genotype A: rtI16T Genotype B: rtR153H Genotype D: rtD134E, rtS135T/Y/N, rtR138N/K, rtN139Q, rtR/W153Q/K^29^, ^30^	Genotype B: rtR153Q, rtA181T Genotype C: rtR153Q, rtA181T, rtM204V, rtL180M+rtM204V, rtV173L+rtL180M+rtM204V 31-33
BCP (1742-1849 nt) and X gene (1374-1838 nt)		Mutations in nucleotide level	Affect binding of BCP to liver-specific transcription factors	Genotype B and C: A1762T+G1764A 24, 34	Genotype B and C: A1762T+G1764A 35 Genotype C: G1613A, C1653T, T1674C, T1753 V, G1764A 36
		Mutations in amino acid level (HBxAg)	Mutant HBxAg may not stimulate CTL activity and favor the clearance of HBsAg	K130M+V131I 24, 34	K130M+V131I 36
preC/C (1814-2452 nt)		Point or double mutation	Mechanism remained exploration but the related mutations are classical risk factors for HBV-related HCC	Genotype C: G1896A (W28*) 34	Genotype B: G1896A+G1899A; Genotype C: A1846T 36

Abbreviations: HBsAg: hepatitis B surface antigen; anti-HBs: antibodies against HBsAg; MHR: major hydrophilic region; RT: reverse transcriptase; BCP: basal core promoter; HBxAg: hepatitis B x antigen; HCC: hepatocellular carcinoma; nt: nucleotide; aa: amino acid; CTL: cytotoxic lymphocyte.

**Table 2:** Prevalence rate, assays and characteristics in HBsAg/anti-HBs coexistence

Prevalence rate		Assays for HBsAg	Assays for anti-HBs	Genotypes in the study	Particular characteristics (compare with HBsAg positive alone group)	Countries	Year of publication
2.4%	39/1606	Architect quantification assays (CMIA, Abbott Laboratories)^ 1^		Genotype B and C	None	China, Wuhan	2015 74
2.6%	145/5513	Quantification on Architect i2000 system (CMIA, Abbott Laboratories)^ 1^ and LIAISON-XL quantification assays (CLIA, Diasorin)^ 2^		Genotype B and C	Lower proportion of patients with HBsAg >250 IU/ml; Lower proportion of patients with HBV DNA > 10^4^ IU/ml	China, Fujian	2017 17
2.9%	122/4169	Elecsys System (ECLIA, Roche Diagnostics)		Genotype B, C and D	Higher genotype D proportion	China, Gansu	2016 75
2.9%	953/32467	Architect quantification assays (CMIA, Abbott Laboratories)^ 1^		Genotype B and C	None	China, Shanghai	2014 23
2.9%	54/1862	AxSYM assay (MEIA, Abbott Diagnostics)		Genotype B and C	Higher anti-HBe positive rate and genotype C proportion	China, Shanghai	2012 30
3.0%	36/1194	Architect quantification assays on Architect i2000 system (CMIA, Abbott Laboratories)^ 1^		Genotype C	Higher anti-HBs level	China, Wuhan	2016 31
3.3%	436/13080	Architect quantification assays on Architect i2000 system (CMIA, Abbott Laboratories)^ 1^		Genotype B and C	Lower proportion of patients with HBsAg >250 IU/ml	China, Jiangsu	2016 16
3.4%	34/1000	Architect quantification assays on Architect i2000 system (CMIA, Abbott Laboratories)^ 1^		Genotype B and C	None	China, Shanghai	2010 21
3.6%	72/1985	Architect quantification assays on Architect i2000 system (CMIA, Abbott Laboratories)^ 1^		Genotype B and C	Lower HBsAg level and HBV DNA concentration	China, Zhejiang	2011 34
4.0%	179/4455	Architect quantification assays on Architect i2000 system (CMIA, Abbott Laboratories)^ 1^		Genotype B and C	None	China, Guangzhou	2018 38
4.9%	20/411	Commercial EIAs by Abbott Laboratories, Roche Diagnostics and Dade Behring		Genotype B2 and C1	Lower serum HBV DNA concentrations	China, Shanghai, Beijing, Guangzhou, Changchun	2007 18
5.8%	505/8687	Cobas E601 quantitative electrochemical luminescence assays (ECLIA, Roche Diagnostics) ^3^		Genotype B and C	Higher genotype C proportion	China, Tianjin	2019 76
1.2%	18/1462	Elecsys system (ECLIA, Roche Diagnostics)	Enzyme-linked immunosorbent assay (ELISA, Diasorin Inc)	Genotype A2, B, C and E	Older median age; lower platelet counts; higher prevalence of HBeAg; lower HBsAg levels	North America	2020 77
2.8%	13/459	AxSYM assay (MEIA, Abbott Laboratories)		Genotype A, B, D, E and A/D	HBV genotypes D was preponderant; HBV DNA levels were significantly higher	France	2007 29
2.9%	353/12191	Elecsys system (ECLIA, Roche Diagnostics), ADVIA Centaur qualitative assays (CLIA, Bayer Diagnostics), and AxSYM assay (MEIA, Abbott Laboratories)		None	Subjects > 50 years old was higher; Higher AST and ALT levels	Korea	2013 78
3.3%	3/92	Enzyme-linked immunosorbent assay (ELISA, Diasorin)		Genotype D	None	Northeastern Brazil	2017 79
5.0%	129/2578	Quantification on Architect i2000 system (CMIA, Abbott Laboratories)^ 1^, Elecsys system (ECLIA, Roche Diagnostics) and LIAISON-XL quantification assays (CLIA, DiaSorin)^ 2^		Genotype B~E and A/F	Lower anti-HBs concentration (<50 IU/L)	France	2015 80
6.4%	48/755	Commercial kits (EIA, Abbott Laboratories)		Genotype C	HBeAg positive rate was higher	South Korea	2009 72
7.0%	73/1042	Commercial assay kits (Abbot Laboratories)		Genotype C	Older median age	South Korea	2014 71
7.1%	166/2341	Quantification on Architect i2000 system (CMIA, Abbott Laboratories)^ 1^		Genotype C	Lower proportion of patients with HBsAg >250 IU/ml	South Korea	2019 53
8.9%	77/866	AxSYM assay (MEIA, Abbott Laboratories)		Genotype A~E	Higher anti-HBs levels	France	2006 14
20.5%	15/73	Radioimmunoassay AUSRIAII-I25 and AUSAB (Abbott Laboratories)		None	Higher serum titers of anti-HBc	Japan	1996 81
21.0%	234/1132	Auszyme II (EIA, Abbott Laboratories)	AxSYM AUSAB (MEIA, Abbott Laboratories)	None	None	Singapore	1996 48

^1^ Architect HBsAg and anti-HBs quantification assays (CMIA, Abbott Laboratories): critical value for positive HBsAg is 0.05 IU/ml, for positive anti-HBs is 10 mIU/mL. ^2^ LIAISON-XL quantification assays (CLIA, Diasorin): critical value for positive HBsAg is 0.05 IU/ml, for positive anti-HBs is 3 mIU/mL. ^3^ Cobas E601 quantitative electrochemical luminescence assays (ECLIA, Roche Diagnostics): critical value for positive HBsAg is 0.05 IU/ml, for positive anti-HBs is 10 IU/L.

## Abbreviations

HBsAg: hepatitis B surface antigen
Anti-HBs: hepatitis B surface antibody
HBV: hepatitis B virus
CHB: chronic hepatitis B infection
HCC: hepatocellular carcinoma
aa: amino acids
CTL: cytotoxic lymphocyte
ORFs: open reading frames
MHR: major hydrophilic region
RT: reverse transcriptase
BCP: basal core promoter
OAS3: oligoadenylate synthetases 3
EIAs: enzyme immunoassays
CIAs: chemiluminescent immunoassays
CLEIA: chemiluminescent enzyme immunoassay
PIVKA-II: proteins induced by vitamin k absence
ER: endoplasmic reticulum
LHB: large hepatitis B surface antigen
MHB: middle hepatitis B surface antigen
SHB: small hepatitis B surface antigen
PKC: protein kinase C
ROS: reactive oxygen species
NA: nucleotide analogues
HBxAg: hepatitis B x antigen
HIV: human immunodeficiency virus
HCV: hepatitis C virus
OBI: occult hepatitis infection
