# Supplementary material for: Paradoxical HBsAg and anti-HBs coexistence among Chronic HBV Infections: Causes and Consequences
Source: Int J Biol Sci. 2021 Mar 11;17(4):1125–37. doi: 10.7150/ijbs.55724 (PMC8040313; doi:10.7150/ijbs.55724)
Supplement: Supplementary file 1 — Supplementary figures and tables. [file ijbsv17p1125s1.pdf]

Supplementary table 1 Mutations associated with HBsAg/anti-HBs coexistence

Regions				Mutation patterns	Mutations appeared in HBsAg/anti-HBs coexistence	Reference
Small HBsAg encoded by S gene	MHR (99-160 aa)	Within 'a' determinant (124-147 aa)	First loop (124-137 aa)	Point mutations	Genotype B: sT126A/S, sG130R/K, sS132P/F, sM133T/S/L, sF134I<br>Genotype C: sT125A/M/H, *sI126S/T*†, sP127T, sA128T/V, sQ129H/K/R/P/S, sG130R/S/A, sT131I/P, M133K/T, sF134L/V/Y/S/I, sP135L, sS136P/F, sC137R/Y	[1-11]
				Glycosylation mutations	Genotype B: sT126N, **sQ129N**, **sG130N**, **sT131N**<br>Genotype C: sI126N, **sQ129N**, **sT131N**, **sG130N+sT131N**, **sG130N+sT131I**, **sT131N+sM133T**	
			Second loop (139-147aa)	Point mutations	Genotype B: sT143L/M, sD144V, ***sG145R***†<br>Genotype C: sC139R/Y, sS/T140I, sK141E, sP142L/S/G/I, sS143M/T, sD144A/E, **sG145R/K/T/G/A**, sN146S, sC147S/R	
		Outside of 'a' determinant (99-123, 148-160 aa)		Point mutations	Genotype B: sM103I, sP105R, sI110L, sS117T, sK122S, sA159G/V<br>Genotype C: sY100S/C, sQ101K/R/H, sL109P, sL110I/S, sP111L, sG112R/E, sT113S, sS114T/A, sT115A/S, sS117G, sT118R, sG119W/R/E, sP120Q/T/A/S, sC121R, sK122R/I, sT123A/V/I, sT148A, sC149R, sP151L, sF158L, sA159G/V	[6, 8, 10, 12]
				Glycosylation mutations	Genotype C: sD99N, sT113N, sS114N, sT115N(C), **sT116N**, sS117N, sK122N, sT123N, sK/R160N	[2, 6-8, 10]
		Inserted mutations			Genotype B: s112-113 "STNR" insertion, s114-115 "S" insertion, s126-127 "SARIVNTT" insertion<br>Genotype C: s112-113"N"/"NG"/"TNR"/"**KNA**" insertion, s114-115 "NT"/"NTT"/"**TTN**"/"**NTSTT**" insertion, s115-116 "**INGTST**" insertions, s116-117 "TTTT"/"STTT"/"STGR" insertions, s127-128 "L" insertion	[2, 3, 8, 10, 13]

		Continuous mutations		Genotype C: sT131N+sS132F+sM133T+sF134I, sT125H+sI126T+sP127T+sA128V	
		Deletions		Genotype C: **sT113N+ deletion in 114-116 aa**, deletion in 117-124 aa	
	Outside of MHR	Close to N-terminal (1-98 aa)	Point mutation	Genotype B: sN3S, sA5S, sL8P, sN40S, sG44E, sS61L, sP67Q, sC76Y, *sI92T*†, *sL94S*†, sV96G/A<br>Genotype C: sT5A, sV14A, sQ16P, sR24K, sP29L, sQ30R, sS31R, sS34L, sN40S, sL42V, sG44E, sP46L, sT47K/A, sP49L/R, sQ51R, sS53L, sS55F, sN59K, sH60P, sP62L, S64F/Y, sC69Y, sW74L, sC76Y, sL77R, sR78Q, sF80S, sI86T, *sI92T*†, *sF93C*†, *sC95W*†, sV96G/A, sL98V	[6, 10, 11, 14, 15]
		Close to C-terminal (161-226 aa)	Point mutation	Genotype B: sY161F, sE164G, sV168A, sL175S, sL186H, sW196L, sM198I, sS204N, sF220C/L/W<br>Genotype C: sF161Y, sE164G/D, sV168A, sS174T, sL175S, sG185R, sP188L, sV190A, sM198W, sP203H, sS204R, sY206C/H, sS210R, sL213I, sF219L/S, sC221Y, sY225F	[6, 8, 10, 14, 16]
			Stop mutations	Genotype B: *sF220stop*†<br>Genotype C: *sC69stop*†, *sW182stop*†, sW199stop, sK212stop, sL216stop, sF219stop	[3, 6, 8, 17]
Large and middle HBsAg encoded by preS/S gene	preS1 (1-119 aa)	4 epitopes for T cells; 9 epitopes for B cells	Deletions	Genotype B: **Deletion in 98-106 aa**<br>Genotype C: **Deletion in 42-47 aa, 57–117 aa, 66-112 aa, 107-113 aa**	[3, 14, 18-21]
			Stop mutations	Genotype C: G59stop, W77stop	
	preS2 (120-174 aa)	2 epitopes for T cells; 1 epitope for B cells	Deletions	Genotype C: **Deletion in 125–135 aa, 122–139 aa, 125–138 aa, 132–141 aa**	
			Stop mutations	Genotype C: R137stop	

		Initiation codon	Initiation codon mutations	Genotype B: **M120I/T/V**	
Polymerase encoded by P gene	RT region (1-344 aa)		Point mutation	Genotype C: rtN13S, rtP17A, rtH55Q, rtS78T, rtP109Q, rtN123I, rtD131E, rtE134V, rtR138K, rtN139Q, rtV142D, **rtR153Q**, rtH156R, rtV173L, rtL180M, **rtA181T**, rtF183stop, rtP184T, **rtM204V**, rtK212stop, rtV214A, rtF221Y; Genotype B: rtR110G, rtN131H, **rtR153Q**/H, **rtA181T**, rtV207M, rtL220V, rtN226T, rtL229M	[1, 3, 9, 11, 22-26]
			Double or multiple mutations	Genotype C: **rtL180M+rtM204V**, **rtV173L+rtL180M+rtM204V**	
BCP (1742-1849 nt) and X gene (1374-1838 nt)			Mutations in nucleotide level	G1742A, **T1753C**, A1755C, **A1762T, G1764A,** T1768A, C1773T , A1775G, T1802C, **A1846T**, ***A1762T+G1764A***†	[3, 10, 26-28]
			Mutations in amino acid level (HBxAg)	**K130M+V131I**	[3, 5, 10]
preC/C (1814-2452 nt)			Point or double mutation in nucleotide level	**G1896A (W28stop), G1899A, *G1896A+G1899A***†	

1. Bold mutations were reported to be associated with an increased risk of HCC.

2. Mutations with the sign (†) and italic format were more frequently discovered in HBsAg/anti-HBs coexistence group compared to HBsAg alone group.

3. Mutations with underline were first found and only occurred in coexistence situation.

4. Abbreviations: HBsAg, hepatitis B surface antigen; anti-HBs, antibodies against HBsAg; MHR, major hydrophilic region; RT, reverse transcriptase; BCP, basal core promoter; HBxAg, hepatitis B x antigen; HCC, hepatocellular carcinoma; nt, nucleotide; aa, amino acid; CTL, cytotoxic lymphocyte.

References

1. Lada O, Benhamou Y, Poynard T, Thibault V. Coexistence of hepatitis B surface antigen (HBs Ag) and anti-HBs antibodies in chronic hepatitis B virus carriers: influence of "a" determinant variants. Journal of virology. 2006; 80: 2968-75.
2. Qiao Y, Lu S, Xu Z, Li X, Zhang K, Liu Y, et al. Additional N-glycosylation mutation in the major hydrophilic region of hepatitis B virus S gene is a risk indicator for hepatocellular carcinoma occurrence in patients with coexistence of HBsAg/anti-HBs. Oncotarget. 2017; 8: 61719-30.
3. Zhou TC, Li X, Li L, Li XF, Zhang L, Wei J. Evolution of full-length genomes of HBV quasispecies in sera of patients with a coexistence of HBsAg and anti-HBs antibodies. Sci Rep. 2017; 7: 661.
4. Wang L, Liu H, Ning X, Gao F. Sequence analysis of the S gene region in HBV DNA from patients positive for both HBsAg and HBsAb tests. Hepatol Res. 2010; 40: 1212-8.
5. Wu IC, Liu W-C, Chang T-T. Applications of next-generation sequencing analysis for the detection of hepatocellular carcinoma-associated hepatitis B virus mutations. J Biomed Sci. 2018; 25: 51.
6. Ding F, Yu HG, Li YX, Cui N, Dai JF, Yu JP. Sequence analysis of the HBV S protein in Chinese patients with coexisting HBsAg and anti-HBs antibodies. J Med Virol. 2015; 87: 2067-73.
7. Yu D-M, Li X-H, Mom V, Lu Z-H, Liao X-W, Han Y, et al. N-glycosylation mutations within hepatitis B virus surface major hydrophilic region contribute mostly to immune escape. Journal of Hepatology. 2014; 60: 515-22.
8. Fu X, Chen J, Chen H, Lin J, Xun Z, Li S, et al. Mutation in the S gene of hepatitis B virus and anti-HBs subtype-nonspecificity contributed to the co-existence of HBsAg and anti-HBs in patients with chronic hepatitis B virus infection. J Med Virol. 2017; 89: 1419-26.
9. Liu W, Hu T, Wang X, Chen Y, Huang M, Yuan C, et al. Coexistence of hepatitis B surface antigen and anti-HBs in Chinese chronic hepatitis B virus patients relating to genotype C and mutations in the S and P gene reverse transcriptase region. Arch Virol. 2012; 157: 627-34.
10. Chen Y, Qian F, Yuan Q, Li X, Wu W, Guo X, et al. Mutations in hepatitis B virus DNA from patients with coexisting HBsAg and anti-HBs. Journal of clinical virology : the official publication of the Pan American Society for Clinical Virology. 2011; 52: 198-203.
11. Colson P, Borentain P, Motte A, Henry M, Moal V, Botta-Fridlund D, et al. Clinical and virological significance of the co-existence of HBsAg and anti-HBs

antibodies in hepatitis B chronic carriers. Virology. 2007; 367: 30-40.

12. Wang J, Zhu B, Lu M, Yang D. Hepatitis B virus preS/S gene mutations and their clinical implications. Annals of Blood. 2017; 2.

13. Liu K, Xie M, Lu X, Yu H, Wang H, Xu Y, et al. Mutations within the major hydrophilic region (MHR) of Hepatitis B virus from individuals with simultaneous HBsAg and anti-HBs in Guangzhou, Southern China. J Med Virol. 2018; 90: 1337-42.

14. Huang X, Qin Y, Zhang P, Tang G, Shi Q, Xu J, et al. PreS deletion mutations of hepatitis B virus in chronically infected patients with simultaneous seropositivity for hepatitis-B surface antigen and anti-HBS antibodies. Journal of medical virology. 2010; 82: 23-31.

15. Mathet VL, Feld M, Espinola L, Sanchez DO, Ruiz V, Mando O, et al. Hepatitis B virus S gene mutants in a patient with chronic active hepatitis with circulating Anti-HBs antibodies. J Med Virol. 2003; 69: 18-26.

16. Zhang JM, Xu Y, Wang XY, Yin YK, Wu XH, Weng XH, et al. Coexistence of hepatitis B surface antigen (HBsAg) and heterologous subtype-specific antibodies to HBsAg among patients with chronic hepatitis B virus infection. Clinical infectious diseases : an official publication of the Infectious Diseases Society of America. 2007; 44: 1161-9.

17. Cento V, Van Hemert F, Neumann-Fraune M, Mirabelli C, Di Maio VC, Salpini R, et al. Anti-HBV treatment induces novel reverse transcriptase mutations with reflective effect on HBV S antigen. The Journal of infection. 2013; 67: 303-12.

18. Zhang D, Dong P, Zhang K, Deng L, Bach C, Chen W, et al. Whole genome HBV deletion profiles and the accumulation of preS deletion mutant during antiviral treatment. BMC Microbiol. 2012; 12: 307.

19. Levrero M, Zucman-Rossi J. Mechanisms of HBV-induced hepatocellular carcinoma. J Hepatol. 2016; 64: S84-s101.

20. Mimms L. Hepatitis B virus escape mutants: "pushing the envelope" of chronic hepatitis B virus infection. Hepatology (Baltimore, Md). 1995; 21: 884-7.

21. Siburian MD, Suriapranata IM, Wanandi SI. Pre-S2 Start Codon Mutation of Hepatitis B Virus Subgenotype B3 Effects on NF-kappaB Expression and Activation in Huh7 Cell Lines. Viral immunology. 2018; 31: 362-70.

22. Yeh C-T. Development of HBV S gene mutants in chronic hepatitis B patients receiving nucleotide/nucleoside analogue therapy. Antivir Ther (Lond). 2010; 15: 471-5.

23. Choi YM, Lee SY, Kim BJ. Naturally occurring hepatitis B virus reverse transcriptase mutations related to potential antiviral drug resistance and liver disease

progression. World J Gastroenterol. 2018; 24: 1708-24.

24. Torresi J, Earnest-Silveira L, Deliyannis G, Edgtton K, Zhuang H, Locarnini SA, et al. Reduced antigenicity of the hepatitis B virus HBsAg protein arising as a consequence of sequence changes in the overlapping polymerase gene that are selected by lamivudine therapy. Virology. 2002; 293: 305-13.

25. Ding F, Miao XL, Li YX, Dai JF, Yu HG. Mutations in the S gene and in the overlapping reverse transcriptase region in chronic hepatitis B Chinese patients with coexistence of HBsAg and anti-HBs. Braz J Infect Dis. 2016; 20: 1-7.

26. Xu H-Z, Liu Y-P, Guleng B, Ren J-L. Hepatitis B Virus-Related Hepatocellular Carcinoma: Pathogenic Mechanisms and Novel Therapeutic Interventions. Gastrointest Tumors. 2014; 1: 135-45.

27. Baptista M, Kramvis A, Kew MC. High prevalence of 1762(T) 1764(A) mutations in the basic core promoter of hepatitis B virus isolated from black Africans with hepatocellular carcinoma compared with asymptomatic carriers. Hepatology (Baltimore, Md). 1999; 29: 946-53.

28. Kuang SY, Jackson PE, Wang JB, Lu PX, Munoz A, Qian GS, et al. Specific mutations of hepatitis B virus in plasma predict liver cancer development. Proc Natl Acad Sci U S A. 2004; 101: 3575-80.
